# Effects of age and reproductive status on individual foraging site fidelity in a long-lived marine predator

**DOI:** 10.1098/rspb.2017.1068

**Published:** 2017-07-26

**Authors:** Stephen C. Votier, Annette L. Fayet, Stuart Bearhop, Thomas W. Bodey, Bethany L. Clark, James Grecian, Tim Guilford, Keith C. Hamer, Jana W. E. Jeglinski, Greg Morgan, Ewan Wakefield, Samantha C. Patrick

**Affiliations:** 1 Environment and Sustainability Institute, University of Exeter, Penryn Campus, Cornwall TR10 9EZ, UK; 2 Centre for Ecology and Conservation, University of Exeter, Penryn Campus, Cornwall TR10 9EZ, UK; 3 Animal Behaviour Research Group, Department of Zoology, University of Oxford, Oxford OX1 3PS, UK; 4 Institute of Biodiversity, Animal Health and Comparative Medicine, University of Glasgow, Graham Kerr Building, Glasgow G12 8QQ, UK; 5 School of Biology, University of Leeds, Leeds LS2 9JT, UK; 6 RSPB Ramsey Island, St David's, Pembrokeshire SA62 6PY, UK; 7 School of Environmental Sciences, University of Liverpool, Nicholson Building, Brownlow Street, Liverpool L69 3GP, UK

**Keywords:** ecology of individuals, foraging, GPS tracking, exploration-refinement foraging hypothesis, seabird, foraging specialization

## Abstract

Individual foraging specializations, where individuals use a small component of the population niche width, are widespread in nature with important ecological and evolutionary implications. In long-lived animals, foraging ability develops with age, but we know little about the ontogeny of individuality in foraging. Here we use precision global positioning system (GPS) loggers to examine how individual foraging site fidelity (IFSF), a common component of foraging specialization, varies between breeders, failed breeders and immatures in a long-lived marine predator—the northern gannet *Morus bassanus*. Breeders (aged 5+) showed strong IFSF: they had similar routes and were faithful to distal points during successive trips. However, centrally placed immatures (aged 2–3) were far more exploratory and lacked route or foraging site fidelity. Failed breeders were intermediate: some with strong fidelity, others being more exploratory. Individual foraging specializations were previously thought to arise as a function of heritable phenotypic differences or via social transmission. Our results instead suggest a third alternative—in long-lived species foraging sites are learned during exploratory behaviours early in life, which become canalized with age and experience, and refined where possible—the exploration-refinement foraging hypothesis. We speculate similar patterns may be present in other long-lived species and moreover that long periods of immaturity may be a consequence of such memory-based individual foraging strategies.

## 1. Introduction

Individual foraging specializations are widespread and occur where animals use a small component of the population niche width [1]. Such inter-individual differences have profound consequences for population dynamics and community structure [1,2], but for long-lived species with delayed maturation, research is biased towards experienced animals, with the significance of individuality in young age classes unknown (but see [3]). This omission may be problematic since age-related differences in other aspects of foraging appear frequently. For example, foraging competency tends to increase with age, with implications for life-history traits [4]. However, the ways in which individual specializations develop as individuals grow towards maturity is unknown.

Individual foraging specializations are generally thought to arise because of heritable phenotypic differences or via social transmission. For example, variation in body-size, jaw or beak morphology may influence diet choice (e.g. [5,6]), and heritable personality differences may covary with differences in foraging location [7]. Alternatively, foraging specializations may be passed on culturally either to offspring (e.g. in sea otters *Enhydra lutris* [8]) or among a wider social group (e.g. in primates [9] and dolphins [10]). In the majority of cases, however, individual specializations arise in the absence of obvious phenotypic differences and independently from conspecifics [1,2]. Therefore, a third alternative explanation is that foraging specializations are learned during individual exploratory behaviours early in life, which then become canalized with age and experience. This may be especially important for some forms of individual specialization such as individual foraging site fidelity (IFSF), where an animal repeatedly uses the same foraging location [11]. Previously, this ‘exploration-refinement’ hypothesis had been proposed to explain inter-individual differences in migratory behaviour of some long-lived animals [12]. By comparing the degree of IFSF between individual birds that vary in terms of constraint and experience, here we provide a first test of this exploration refinement foraging hypothesis.

Seabirds represent an excellent group to study how individual specialization varies with age and reproductive status. First, individual specialization appears common among adult seabirds—approximately 87% of studies found evidence of specialization in terms of foraging or feeding, particularly IFSF [11,13–16]. Such consistent behaviours are likely to be linked with the predictability of marine resources where, particularly in sub-polar, temperate, neritic and frontal waters, oceanographic features create prey patches that are persistent in both time and space, favouring learning and hence IFSF [17]. Second, seabirds have bet-hedging life-history strategies with long periods of immaturity [18]. This period is important for the development of effective foraging, particularly for finding patchy prey distant from land [18–20]. However, it is unknown whether IFSF may also play a role during this period of development. Third, many seabirds are large enough to carry bio-logging devices without major impacts upon behaviour, making it possible to reconstruct individual spatial foraging behaviour in fine detail, and precisely reveal the degree of IFSF.

Here we focus on IFSF in northern gannets *Morus bassanus* (hereafter ‘gannets’), large (approx. 3 kg), long-lived (annual survival probability approximately 92%), medium-ranging (100 s of km) colonial-nesting seabirds that breed along the coasts of temperate and boreal waters in the North Atlantic [21,22]. Longitudinal research reveals adult gannets have highly consistent individual differences in foraging behaviours including searching [13], site fidelity [11,15], route fidelity [15,16] and diet [14]. Moreover, IFSF is repeatable both within and among years indicating this does not simply reflect short-term differences in prey gain, such as would be expected by, for example, a win–stay, lose–shift strategy [11–15]. Gannets do not breed for the first time until they are a minimum of 4–5 years old [21]. Inexperienced immatures either engage in prospecting or become central place foragers during the breeding season [23], but nothing is known about foraging individuality.

We use high-precision global positioning system (GPS) telemetry to compare IFSF of gannets in three groups: (i) successful breeders, (ii) failed breeders (experienced birds not constrained by chick rearing) and (iii) immatures, all tracked over successive foraging trips. Specifically, we compare IFSF in terms of foraging locations (distal point of trips) and foraging route fidelity, as well as foraging effort (distance travelled). If immatures have lower IFSF than adults, this would provide support for the exploration refinement foraging hypothesis. Moreover, by tracking failed breeders we can also better understand the potentially confounding influence of reproductive constraint. If the degree of IFSF were similar between breeders and immatures, this would indicate some other mechanism at play.

## 2. Material and methods

### (a) Study site and device deployment

Fieldwork was conducted on an uninhabited island, Grassholm, Wales, UK (51°43′ N, 05°28′ W), during July/August 2010, 2015 and 2016 where approximately 40 000 pairs of gannets breed alongside several thousand immatures [23].

To compare at-sea behaviour among birds of different age classes and reproductive status we tracked 15 immatures (8 females, 7 males; aged 2–3 years), 46 chick-rearing adults (15 females, 31 males; aged more than 5 years; hereafter ‘breeders’) and five individually marked adults that had bred successfully in the past but had failed by the time of capture (while it is possible these birds may have been taking a sabbatical year, this sort of breeding deferral is unknown in gannets [21]; two females, three males; aged more than 5 years; hereafter ‘failed breeders’). Immatures were caught (using a metal crook attached to an approximately 5 m carbon fibre pole) while attending non-breeding aggregations at the colony periphery, and approximately aged (up to 5 years, although we focused only on 2–3-year-olds) on the basis of plumage and bare parts [21]. They were fitted either with a 40 g GPS-platform terminal transmitter in 2010 (GPS-PTT; Microwave Telemetry), a 38 g GPS global system for mobile communications tag in 2015 (GPS-GSM; Ecotone, Poland) or a 35 g GPS-GSM tag in 2016 (Nanofix; Pathtrack) attached using Tesa tape and, for the GPS-PTTs, steel lock cable ties (Ty*-*Rap) to the tail, representing 1.2–1.4% of immature body mass (2857 g ± 167.5). Breeders and failed breeders were caught at the nest on changeover (using the same metal crook), targeting the departing bird to ensure any chicks were not left unattended and/or to ensure a foraging trip began immediately upon release. In 2010 birds were fitted with a 30 g I-gotU GPS logger (GT200 or GT600, Mobile Action Technology) fitted to the tail using Tesa^®^ tape and in 2015/16 a 20 g GPS logger (GT120) fitted in the same way along with a 16 g altimeter (the altimeter data not included in this study). These devices represent 1.0–1.2% of breeder/failed breeder body mass (3010 g ± 284.1). Despite the difference in percentage logger mass deployed on immatures and adults, we think it extremely unlikely that such a small difference (less than 0.5% of body mass) would have any detectable effect on the foraging behaviour studied here. GPS-PTTs were programmed to obtain a GPS fix hourly, relayed via the Argos satellite system every 48 h. GPS-GSM tags took a fix every 30 min (Ecotone) or a maximum of 5 min (Pathtrack), relayed via the mobile phone network. I-gotU GPS loggers were programmed to obtain a fix every two minutes, with data downloaded upon bird recapture and device retrieval. We took approximately 0.1 ml of blood from the tarsal vein for molecular sexing (at the University of Exeter or commercially outsourced to AvianBiotech.com).

### (b) Analysis of tracking data

To allow comparison of GPS tracking data from immatures (GPS-PTT: 85% hourly fixes 15% every two hours; GPS-GSM: fixes between 5 and 30 min) and adults (100% of fixes every two minutes), we first filtered the data to remove erroneous fixes as indicated by unrealistic flight speeds [24]. Next, all tracking data were sub-sampled to ensure a resolution of one fix per hour.

Immature movements can be broadly divided into two states: (i) central place foraging and (ii) prospecting [23]. Prospecting has a very different function from central place foraging [25], therefore we removed all prospecting trips from immatures [26] and from one breeder.

To compare IFSF between immatures, breeders and failed breeders, we calculated three indices from complete foraging trips: (i) foraging route fidelity, (ii) foraging site fidelity and (iii) foraging effort.

#### (i) Foraging route fidelity

We calculated individual route fidelity using nearest-neighbour distance (NND, in km). This technique quantifies the spatial similarity between a focal trip and comparison trip by calculating the distance from each location along a track to its nearest neighbour on the comparison track [27]. The NND calculated between two trips decreases with the spatial similarity. NND was calculated in two ways: (i) for all within individual trips (i.e. a measure of route fidelity across all repeat trips of the same bird) and (ii) for all among-individual trips for breeders, failed breeders and immatures separately (i.e. a comparison of route fidelity within all individuals of the same age class). Locations less than 2 km from the colony were excluded because gannets often gather on the water here in non-foraging rafts [26].

We used linear mixed models (LMM) to assess whether route fidelity varied significantly between the three groups. We compared within-individual NND to among-individual NND for breeders, failed breeders and immatures separately, as well as comparing within-individual NND among all three groups, including sex and year as fixed effects, and pair as a random effect, and comparing each model with the null (intercept only) model based upon likelihood-ratio tests (LRTs). NND was natural-square-root-transformed to obtain normality. To account for differences in NND due to differences in trip length, we added the difference in trip length between each pair of trips compared as a covariate. In total we used 152 trips from 46 breeders, 15 trips from 5 failed breeders and 70 trips from 15 immatures.

#### (ii) Foraging site fidelity

As a measure of foraging site fidelity we first estimated the distal point (longitude and latitude) of each foraging trip and then compared the similarity of these values between repeat distal locations both within and among individuals. Even though gannets may forage throughout the course of a foraging trip, distal location is considered an appropriate measure of IFSF as a high percentage of dives occur at the furthest point from the colony [28,29].

#### (iii) Foraging effort

We calculated two measures of foraging effort: (i) total distance travelled (km) and (ii) distance to distal point (furthest distance from the colony, km).

We compared individual consistency of foraging site fidelity and foraging effort among immatures, breeders and failed breeders by calculating repeatability (*r*) for each group using the rptR package in R [30], with sex included as a fixed effect in all models, and year included as a fixed effect where multiple years are present (0 = low repeatability, 1 = high repeatability). Repeatability of total distance travelled and maximum distance from the colony were both transformed using Box–Cox transformations.

## 3. Results

### (a) Foraging trips

**Figure 1.**
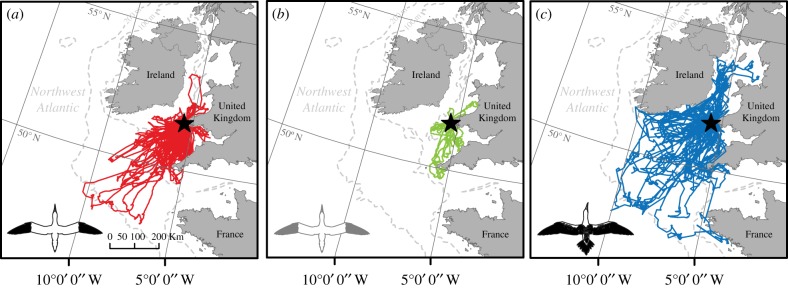
Foraging movements of breeding, failed breeding and immature gannets. Central-place foraging trips reconstructed from GPS tracked birds from Grassholm, UK (July/August 2010, 2015 and 2016). (*a*) Chick-rearing birds aged 5+ years (*n* = 46 individuals, 152 trips; median 3 trips per individual), (*b*) failed breeders aged 5+ years (*n* = 5 individuals, 15 trips; median 3 trips per individual) and (*c*) immatures aged 2–3 years (*n* = 15 individuals, 70 trips; median 4 trips per individual). (Online version in colour.)

During July/August 2010, 2015 and 2016 we obtained central placed movements from 15 immatures, 5 failed breeders and 46 breeders. For the breeders, we GPS-tracked 152 complete foraging trips, a median of 3 repeat trips per bird (range 2–8 trips per bird; trip duration 84–4504 min; total distance travelled 11.6–1246.1 km and maximum distance from the colony 10.8–516.7 km; figure 1; electronic supplementary material, figure S2). For immatures, we obtained 70 foraging trips, with a median of 4 repeat trips per bird (range 2–8 trips per bird; trip duration 34–16 470 min; total distance travelled 4.2–2216.6 km and maximum distance from the colony 2.1–5538.0 km; figure 1; electronic supplementary material, figure S3). For the failed breeders, we obtained 15 foraging trips with a median of 3 repeat trips per bird (range 2–4 trips per bird; trip duration 136–2898 min; total distance travelled 47.7–436.8 km and maximum distance from the colony 23.9–184.7 km; figure 1; electronic supplementary material, figure S4).

### (b) Foraging route fidelity

**Figure 2.**
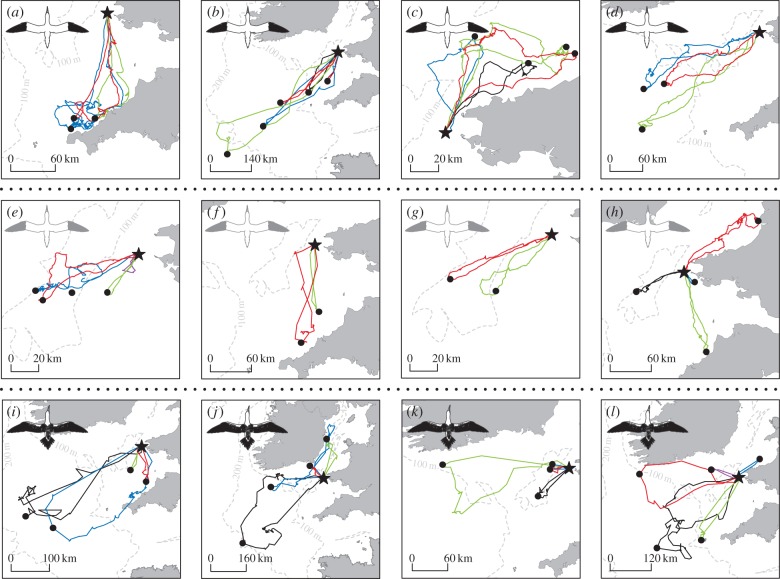
Individual foraging site fidelity differs between breeding, failed breeding and immature gannets (Grassholm, UK; July/August 2010, 2015 and 2016). Distal locations are marked with a black dot. Top row (*a*–*d*) shows 3–5 trips from 4 breeders, illustrating the high degree of individual foraging site and route fidelity. Middle row (*e*–*h*) shows 2–4 trips for 4 failed breeders, with (*e*–*g*) showing strong site fidelity, but (*h*) showing more exploratory movements. Bottom row (*i*–*l*) shows 4–5 trips from immatures illustrating low levels of individual foraging site and route fidelity. The individual trips have been selected for illustration purposes—all trips are shown in electronic supplementary material, figures S2–S4. Trips are colour-coded chronologically: 1 = red; 2 = black; 3 = green; 4 = blue; 5 = magenta. (Online version in colour.)

**Figure 3.**
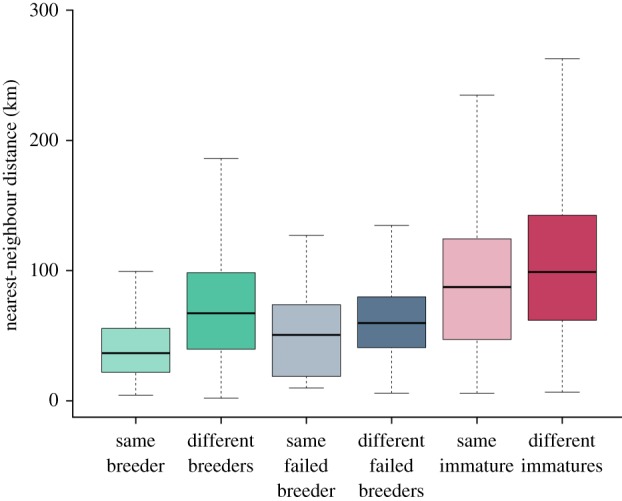
Nearest-neighbour distance (NND mean ± s.d. in km) reveals that within-individual route fidelity varies by age and reproductive state in gannets. Adults show within-individual route fidelity and immatures do not, while failed breeders are intermediate. (Online version in colour.)

NNDs of breeders showed that repeat foraging routes were more similar within than among individuals (LRT: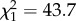, *p* < 0.001, *R*^2^ = 0.68; table 1, figures 2 and 3). In contrast, for immatures, variation in NND within individuals was similar to variation among individuals (LRT: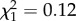, *p* = 0.729, *R*^2^ = 0.42; table 1, figures 2 and 3). This was also the case for failed breeders (LRT: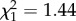, *p* = 0.230, *R*^2^ = 0.42). A comparison of NNDs between age classes revealed that there were significant differences in within-individual route consistency among age groups (LRT: 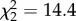, *p* < 0.001, *R*^2^ = 0.67). Routes followed by different individuals of the same group were as dissimilar among all groups (LRT: 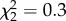, *p* = 0.853, *R*^2^ = 0.63). Trip duration, year and sex were all retained in the analysis, but, given they are beyond the focus of the study, are not discussed further (electronic supplementary material, table S1 and figure S1).

**Table 1.** Nearest-neighbour distance (NND) reveals within-individual route fidelity varies by age in gannets. Adults show within-individual route fidelity, whereas immatures and failed breeders do not.

age group	mean NND ± s.e. (km)	pairs of trips	individuals (trips)
adults (within individual)	49.4 ± 3.1	214	46 (152)
adults (among individuals)	73.9 ± 0.4	10 961	46 (152)
failed breeders (within individuals)	55.2 ± 9.8	17	5 (15)
failed breeders (among individuals)	62.1 ± 3.5	88	5 (15)
immatures (within individual)	95.7 ± 4.7	165	15 (70)
immatures (among individuals)	107.1 ± 1.3	2250	15 (70)

### (c) Foraging site fidelity

During repeat trips breeders had highly repeatable distal latitude and longitude (table 2, figure 2*a*–*d*). In contrast, immatures showed highly variable distal locations (table 2, figure 2*i*–*l*). Failed breeders were intermediate (figure 2*e*–*h*), having highly repeatable distal longitudes, but not latitude—although the majority of tracked birds showed very similar foraging locations (table 2, figure 2*e*–*h*; electronic supplementary material, figure S3).

**Table 2.** Repeatability (*r* ± s.e., with 95% CIs in parentheses) of gannet foraging site fidelity (decimal degrees) and foraging effort (0 = low repeatability, 1 = high repeatability). Breeders showed repeatable foraging sites (distal longitude and latitude), in contrast to immatures, which showed highly variable foraging sites. Failed breeders showed repeatable foraging longitudes, but not latitude with much variation. Foraging effort showed low repeatability for all groups. Significantly repeatable foraging behaviours are given in italics.

	breeders (*n* = 46)	failed breeders (*n* = 5)	immatures (*n* = 15)
foraging site fidelity			
longitude of distal point (DD)	*0.51 ± 0.08* (*0.33, 0.66*)	0.42 ± 0.27 (0, 0.82)	0.00 ± 0.07 (0, 0.24)
latitude of distal point (DD)	*0.34 ± 0.09* (*0.14, 0.51*)	0.00 ± 0.17 (0, 0.56)	0.00 ± 0.07 (0, 0.24)
foraging effort			
total distance travelled (km)	0.11 ± 0.08 (0, 0.29)	0.01 ± 0.18 (0, 0.61)	0.00 ± 0.07 (0, 0.25)
distance to distal point (km)	0.15 ± 0.08 (0, 0.31)	0.00 ± 0.17 (0, 0.56)	0.00 ± 0.07 (0, 0.22)

### (d) Repeatability of foraging effort

Analysis of within-individual variation showed that foraging effort (total distance travelled and distance to distal point) was not repeatable, regardless of group (table 2).

## 4. Discussion

Our study shows clear differences in IFSF between breeding and immature gannets. Breeders returned repeatedly to similar locations, using similar routes during consecutive foraging trips (figures 2 and 3). By contrast, immatures tracked over the same period showed little or no evidence of IFSF, with highly variable distal points and low levels of route fidelity (figures 2 and 3). Both age groups had similarly very low repeatability in terms of foraging effort. Failed breeders were somewhat intermediate—some individuals showed strong site fidelity, while others were less repeatable (figures 2 and 3). The potential causes of these differences, as well as their implications for life history, conservation and the development of individual foraging specializations, are discussed below.

### (a) Individual foraging site fidelity in breeders

Individual specializations may be ubiquitous among marine vertebrates, with consistent foraging behaviours reported across a diversity of wide-ranging taxa including cartilaginous fish [31], bony fish [32], reptiles [33], mammals [34] and seabirds [13,16]. These predators are probably responding to the generally predictable distribution of marine prey, but long-term IFSF shows this is not simply a response to short-term opportunities [11–15]. Gannets, for example, return repeatedly to the same sites characterized by persistent ocean fronts [11,35] or consistently high fishing activity [36]. The high degree of IFSF exhibited by breeders here contrasts with low repeatability in foraging effort, which may relate to variation in transit costs because of wind [37] or visibility, or fluctuations in individual energetic demands. Nevertheless, site fidelity varied within individuals (electronic supplementary material, table S1), revealing that foraging sites are not absolute.

### (b) Individual foraging site fidelity in immatures and failed breeders

In contrast to breeders, immatures showed low IFSF—they had highly variable routes and distal points varied over time (figures 2 and 3). This difference may arise for a number of reasons. First, as predicted by the exploration refinement foraging hypothesis, these differences may relate to learning. Site familiarity could be attained via individual exploration [12] or social information use [28,38] early in life, with acquired navigational memory canalizing such behaviours [12]. Immature gannets perform directed commutes followed by area-restricted searches [23], and such searching is learned rapidly in post-fledging albatrosses [19]. However, our data suggest that knowledge of site fidelity may take much longer to accrue. Second, the magnitude of individual foraging specialization may be positively correlated with intra-specific competition [2,39], with longer immature foraging trips producing lower conspecific densities compared with breeders. While we cannot completely exclude this possibility, we think it is unlikely for the following reasons: (i) immature gannets also make many short trips, being exposed to similar levels of competition experienced by breeders (figure 2; electronic supplementary material, figure S3); (ii) during long trips, immatures share foraging grounds with adults from adjacent colonies [38], exposing them to high levels of inter-colony competition; and (iii) a comparison of adult IFSF among seven gannet colonies revealed no relationship with colony size (T.W.B. *et al.* 2017, unpublished). Therefore, while intra-specific competition could be an important driver of foraging site fidelity in some taxa [1,2], the current evidence suggests this is unlikely for gannets. Third, differences between breeders and immatures may arise because of differences in habitat predictability (figure 1). We think this is an unlikely explanation for the differences among age groups, however, since all birds forage in water masses characterized by highly productive and predictable oceanographic conditions [40]. Fourth, IFSF may arise because reproduction imposes strong time and energetic constraints, reducing opportunities for exploratory movements [11]. We believe that reproductive constraints are important, but are not the primary cause of age-specific differences in IFSF. While immatures had longer trips overall (figures 1 and 2), ranges overlap considerably and differences in NND remain despite including trip duration as a covariate. Hence, if there is not an experience-based difference in IFSF between immatures and breeders when trips are of similar length, then breeders should explore in much the same way that immatures do, which is not the case. Furthermore, the majority of failed breeders were highly site faithful, (figure 2*e*–*h*; electronic supplementary material, figure S4), in the absence of breeding constraints. We therefore conclude the observed patterns are best explained by differences in experience, with young birds yet to learn the whereabouts of suitable foraging sites. We also note that since IFSF is not absolute, exploration and refinement may continue throughout life, with exploratory movements being more likely, and likely to occur more often, when not constrained by breeding.

### (c) Age-specific variation in foraging individuality

Age-specific differences in avian foraging are not uncommon [4], but our study is, as far as we know, the first to demonstrate differences in IFSF. The ability to find sufficient food for both self-maintenance and reproduction is believed to influence age at first breeding in many long-lived species [41], and is also related to the positive correlation between foraging efficiency and age [20]. Based on our findings, we propose that individual foraging specialization may also play an important, previously overlooked role in age-specific foraging. Further work should examine the relationship between individual foraging specialization and age at first breeding, as well as revealing more about refinement of individuality beyond age at first breeding.

### (d) Wider implications

Individuality in behavioural traits has wide-reaching applied implications [2], as do the age-related differences in foraging specialization reported here. Conservation biologists recognize the importance of foraging individuality in terms of maintaining diversity and risk management [1]. For example, variation in individual specialization may mean adults and immatures have different levels of risk from fisheries bycatch [14] or collision with marine renewables.

## 5. Conclusion

Our study found that breeding gannets had individually consistent foraging routes and sites, failed breeders were less consistent, while immatures tended to switch between different sites and routes during successive trips. Since IFSF is probably driven by site familiarity [11], the age-specific differences reported here are probably best explained by age-specific differences in experience. We conclude that immature seabirds are likely to be accruing experience of suitable foraging sites, which become canalized later in life, as posited by the exploration-refinement foraging hypothesis. We also hypothesize that energetic and time constraints imposed by reproduction may shape opportunities for exploration (and therefore IFSF), and moreover that foraging refinement probably continues throughout an animal's lifetime. More work is needed to understand whether age-specific variation in individual foraging occurs in other long-lived species and whether it plays a role in key life-history characters such as age at first breeding.

## Supplementary Material

Table of tracking data; Plots of NND sex differences; Breeder tracking data; Immature tracking data; Failed breeder tracking data

## Data accessibility

Data are available via Dryad (http://dx.doi.org/10.5061/dryad.8m1nf) [42].
