# Supplementary material for: Effects of age and reproductive status on individual foraging site fidelity in a long-lived marine predator
Source: Proc Biol Sci. 2017 Jul 26;284(1859):20171068. doi: 10.1098/rspb.2017.1068 (PMC5543227; doi:10.1098/rspb.2017.1068)

Electronic supplementary material, Table 1. Tracking data including in final analysis. Including here, the coefficient of relative plasticity (CRP), calculated for each individual as the ratio of the individual variance over the overall variance for each group. 0 = highly repeatable, 1 = low repeatability.

				**Coefficient of Relative Plasticity**				
**Individual**	**Sex**	**#trips**	**year**	**Term. lat**	**Term. long**	**Term. dist.**	**Total dist.**	**Trip duration**
**Immatures (n=15)**								
57619	female	4	2010	0.007	0.166	0.077	0.053	N/A
57620	female	4	2010	0.608	0.248	0.237	0.244	N/A
57621	female	4	2010	0.606	0.330	0.178	0.205	N/A
57628	female	4	2010	0.269	0.196	0.379	0.325	N/A
57631	female	4	2010	0.410	0.024	0.403	0.777	N/A
57633	female	4	2010	0.098	0.388	0.228	0.280	N/A
57634	male	4	2010	0.417	0.266	0.242	0.292	N/A
57643	male	2	2010	1.000	1.000	1.000	1.000	N/A
G394	male	4	2015	0.007	0.042	0.017	0.034	0.094
G395	female	5	2015	0.137	0.291	0.107	0.151	0.362
G401	male	3	2015	0.010	0.117	0.042	0.057	0.094
G407	female	4	2015	0.173	0.047	0.090	0.086	0.190
G429	male	5	2016	0.053	0.140	0.029	0.112	0.266
G430	male	7	2016	0.088	0.159	0.111	0.329	1.000
G433	male	8	2016	0.102	0.153	0.072	0.113	0.302
**Mean (±SE)**				**0.266 (± .069)**	**0.238 (±0.057)**	**0.214 (±0.059)**	**0.201 (±0.066)**	**0.329 (±0.102)**
**Failed breeders (n=5)**								
G071	male	3	2015	0.104	0.002	0.041	0.020	0.012
G075	male	4	2015	0.052	0.080	0.020	0.010	0.009
G135	male	2	2015	0.000	0.010	0.002	0.000	0.000
G278	female	2	2015	0.009	0.002	0.009	0.013	0.029
G323	female	4	2015	0.000	0.009	0.003	0.002	0.001
**Mean (±SE)**				**0.033 (±0.02)**	**0.021 (±0.015)**	**0.015 (±0.001)**	**0.009 (±0.004)**	**0.010 (±0.005)**
**Breeders (n=46)**								
G004	male	4	2010	0.025	0.111	0.032	0.040	0.021
G006	male	4	2010	0.017	0.051	0.028	0.019	0.008
G009	male	3	2010	0.001	0.004	0.001	0.013	0.020
G010	male	3	2010	0.035	0.011	0.035	0.038	0.018
G011	male	5	2010	0.121	0.187	0.216	0.116	0.027
G013	male	3	2010	0.008	0.035	0.015	0.007	0.000
G015	female	4	2010	0.002	0.015	0.008	0.007	0.007
G016	male	4	2010	0.000	0.001	0.001	0.001	0.003
G019	female	8	2010	0.052	0.051	0.078	0.039	0.045
G022	female	3	2010	0.017	0.005	0.013	0.007	0.004
G024	female	3	2010	0.054	0.008	0.063	0.028	0.011
G025	male	3	2010	0.010	0.012	0.014	0.001	0.000
G026	male	3	2010	0.020	0.008	0.021	0.010	0.002
G027	male	4	2010	0.000	0.021	0.003	0.005	0.009
G030	male	6	2010	0.248	0.163	0.315	0.187	0.038
G031	female	4	2010	0.088	0.017	0.083	0.052	0.009
G032	male	4	2010	0.046	0.041	0.036	0.021	0.008
G033	male	3	2010	0.046	0.041	0.039	0.023	0.000
G037	male	3	2010	0.011	0.002	0.012	0.007	0.008
G038	male	4	2010	0.002	0.002	0.001	0.003	0.004

G359	female	2	2015	0.001	0.000	0.001	0.002	0.001
G368	male	2	2015	0.002	0.004	0.003	0.001	0.000
G372	male	2	2015	0.097	0.099	0.004	0.001	0.003
G374	male	2	2015	0.000	0.027	0.008	0.023	0.018
G376	male	2	2015	0.014	0.004	0.000	0.002	0.003
G379	male	2	2015	0.001	0.000	0.000	0.000	0.004
G385	female	3	2015	0.001	0.020	0.008	0.004	0.003
G386	male	3	2015	0.005	0.002	0.005	0.005	0.006
G388	male	3	2015	0.021	0.007	0.022	0.012	0.004
G413	female	2	2016	0.005	0.003	0.004	0.001	0.000
G415	male	4	2016	0.032	0.001	0.031	0.013	0.007
G424	female	2	2016	0.002	0.010	0.007	0.002	0.003
G434	male	2	2016	0.066	0.001	0.046	0.026	0.013
G435	female	2	2016	0.039	0.018	0.046	0.040	0.080
G441	male	2	2016	0.042	0.022	0.048	0.027	0.008
G444	male	2	2016	0.000	0.000	0.000	0.000	0.000
G446	female	2	2016	0.007	0.013	0.005	0.002	0.002
G447	male	2	2016	0.000	0.003	0.000	0.001	0.004
G450	female	2	2016	0.005	0.035	0.011	0.006	0.010
G451	female	2	2016	0.002	0.009	0.006	0.003	0.003
G454	female	2	2016	0.017	0.006	0.001	0.001	0.000
G458	female	2	2016	0.000	0.001	0.000	0.003	0.023
G461	male	4	2016	0.004	0.017	0.011	0.010	0.008
G464	male	3	2016	0.016	0.012	0.016	0.016	0.050
G466	male	2	2016	0.002	0.000	0.002	0.002	0.002
G469	male	5	2016	0.005	0.004	0.003	0.005	0.005
**Mean (±SE)**				**0.026 (±0.006)**	**0.024 (±0.001)**	**0.028 (±0.008)**	**0.018 (±0.004)**	**0.011 (±0.002)**

Electronic supplementary material, Figure 1. Plots of NND (mean±SE) for gannets tracked during multiple trips. (a) Within-individual route similarity for each group, by sex and (b) among-individuals only, for each group, by sex. Different females followed less similar routes than different males, in both breeders and immatures, and females also showing less route fidelity than males (at least in immatures, although less clear in adults).

(a)

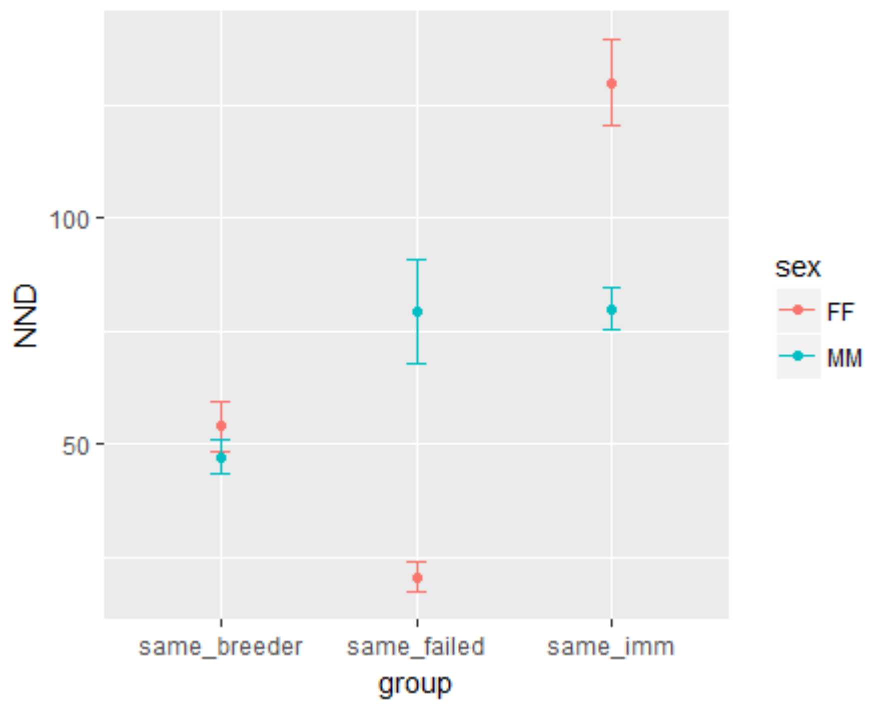

(b)

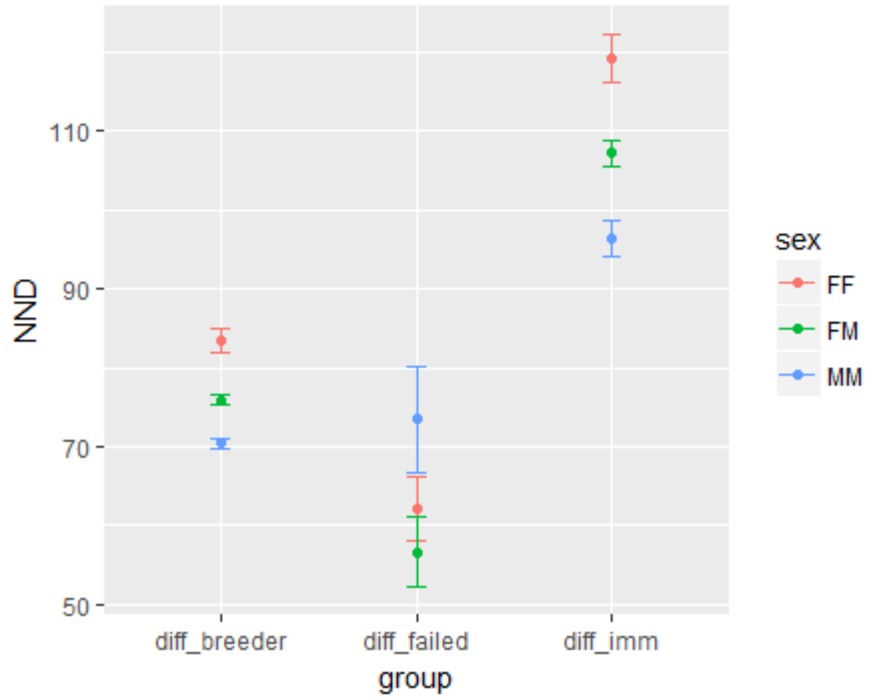

Electronic supplementary material, Figure 2. Repeat foraging tracks of (*n*=46) chick-rearing northern gannets from Grassholm, UK July/August 2010, 2015 and 2016 generated from GPS loggers on a 2 minute duty cycle. Tracks are colour-coded by number: track 1=black; track 2=blue; track 3=red; track 4 = green; track 5 = purple; track 6=yellow. Data is presented for 15 females and 31 males.

G006 - breeding male (2010, n = 4 trips)

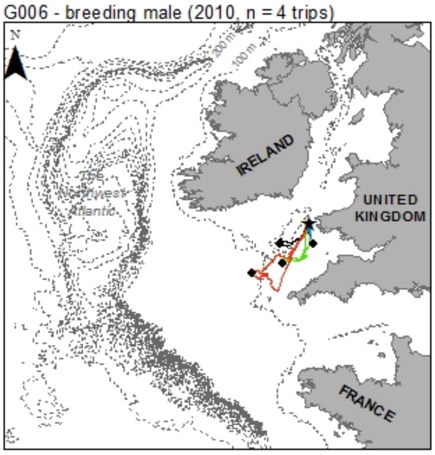

G004 - breeding male (2010, n = 4 trips)

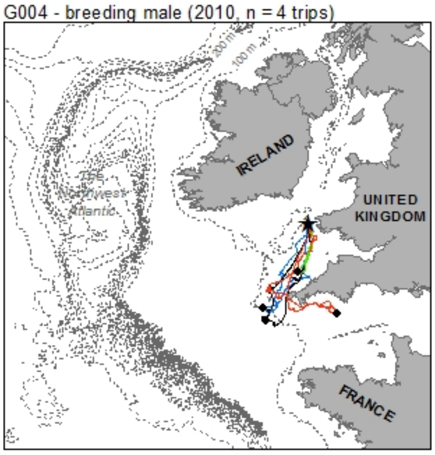

G009 - breeding male (2010, n = 3 trips)

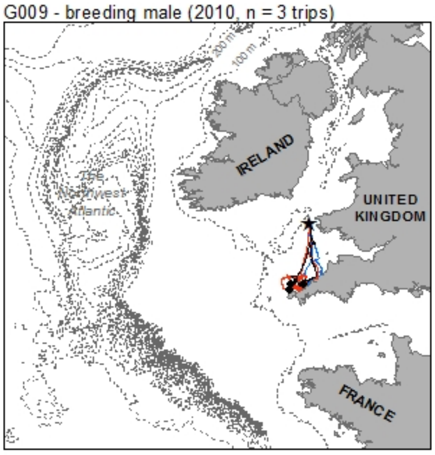

G010 - breeding male (2010, n = 3 trips)

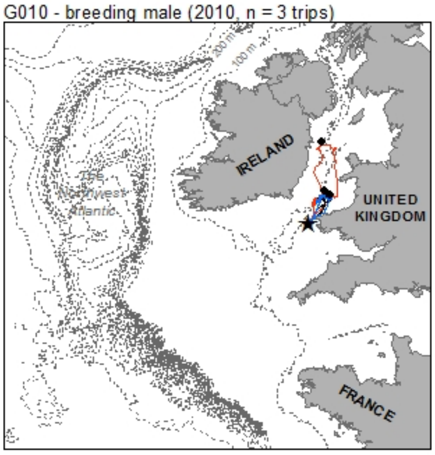

G016 - breeding male (2010, n = 4 trips)

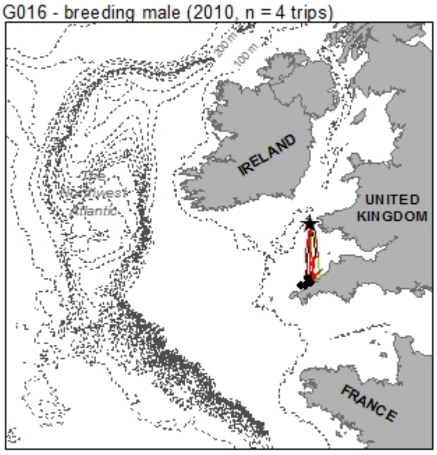

G013 - breeding male (2010, n = 3 trips)

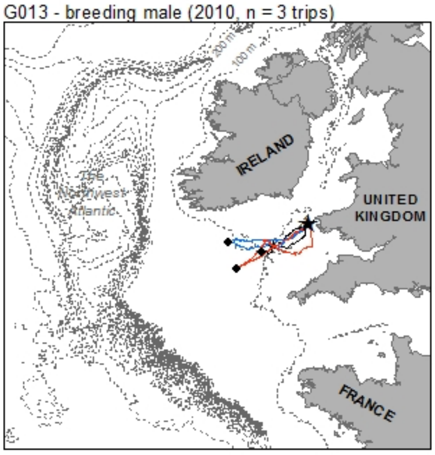

G025 - male (2010, n = 3 trips)

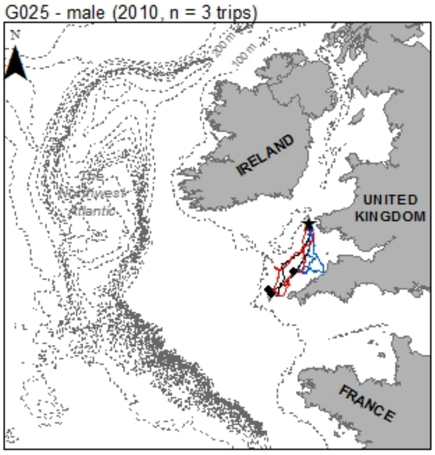

G026 - male (2010, n = 3 trips)

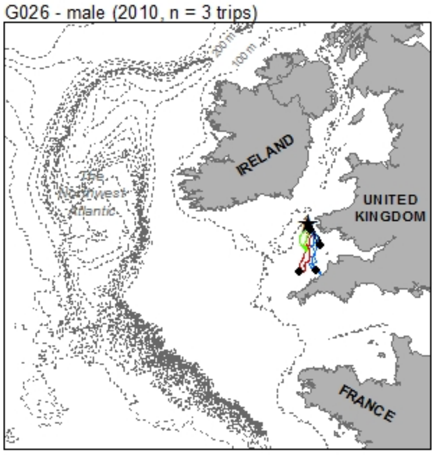

G027 - male (2010, n = 4 trips)

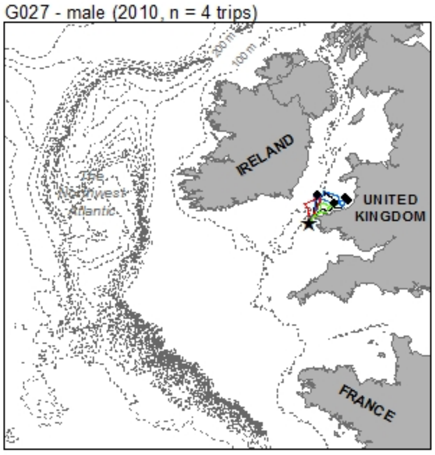

G030 - male (2010, n = 6 trips)

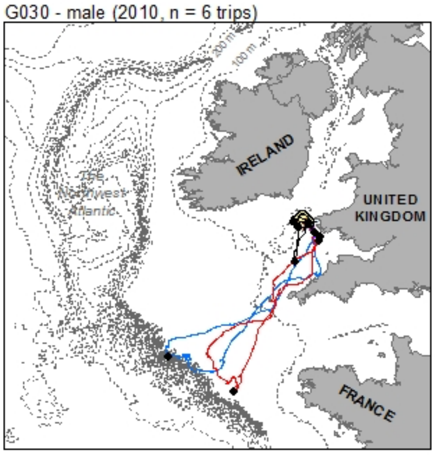

G032 - male (2010, n = 4 trips)

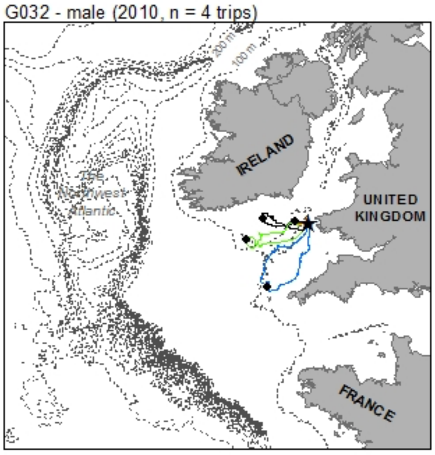

G033 - male (2010, n = 3 trips)

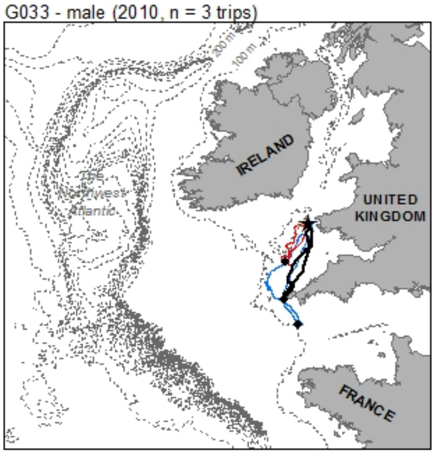

G015 - breeding female (2010, n = 4 trips)

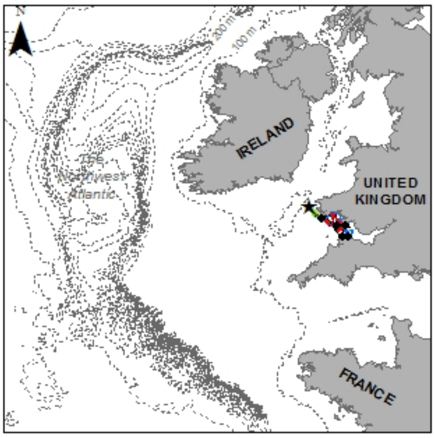

G022 - breeding female (2010, n = 3 trips)

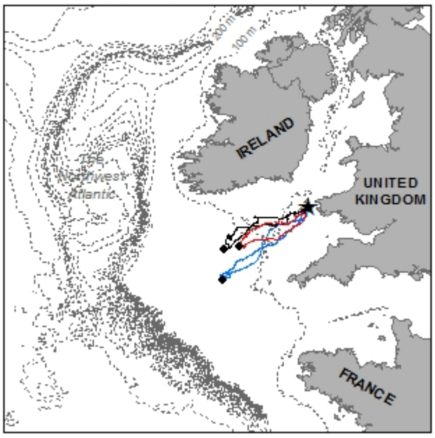

G024 - breeding female (2010, n = 3 trips)

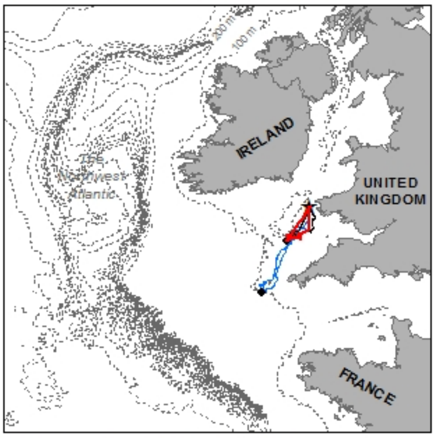

G031 - breeding female (2010, n = 4 trips)

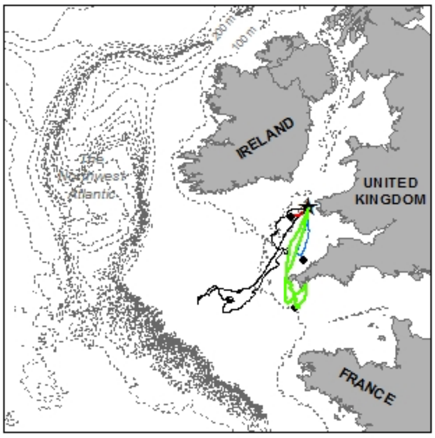

G037 - breeding male (2010, n = 3 trips)

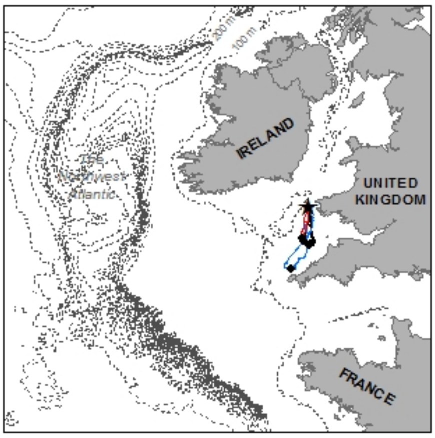

G038 - breeding male (2010, n = 4 trips)

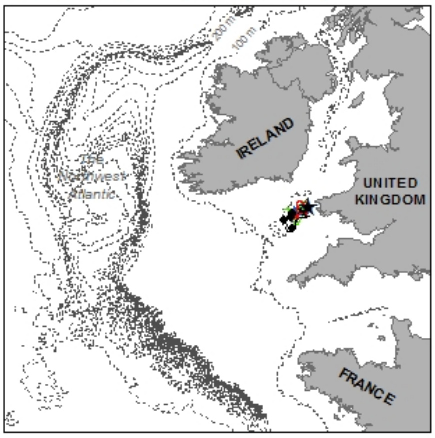

G376 - breeding male (2015, n = 2 trips)

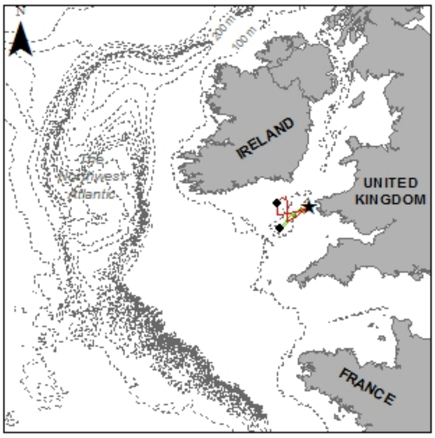

G379 - breeding male (2015, n = 2 trips)

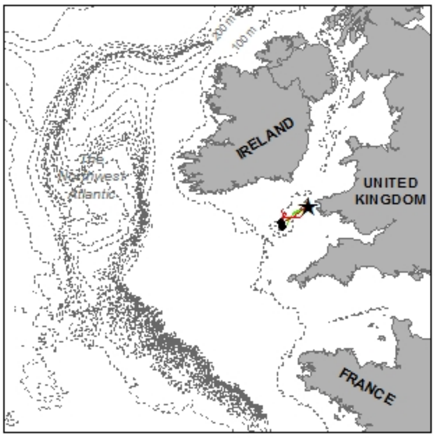

G385 - breeding female (2015, n = 3 trips)

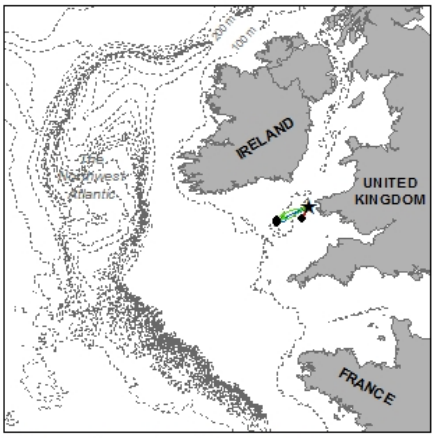

G386 - breeding male (2015, n = 3 trips)

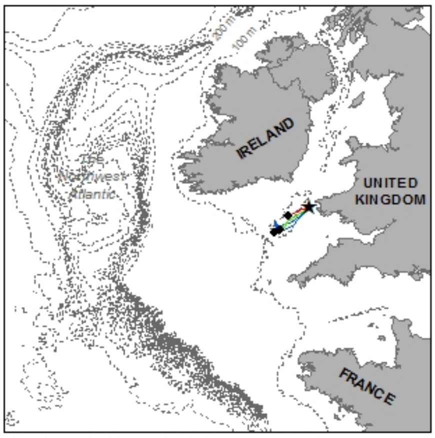

G388 - breeding male (2015, n = 3 trips)

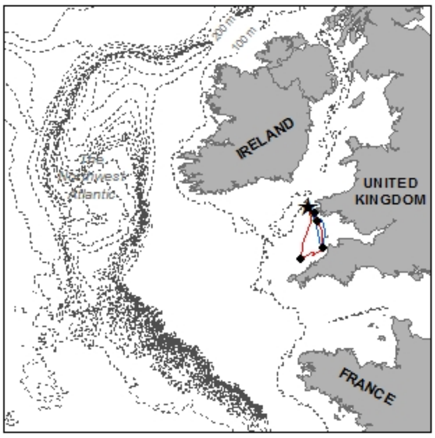

G374 - breeding male (2015, n = 2 trips)

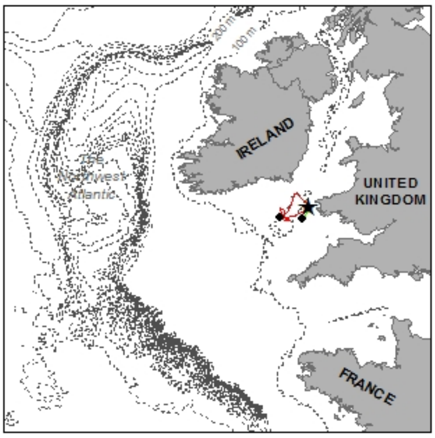

G413 - breeding female (2016, n = 2 trips)

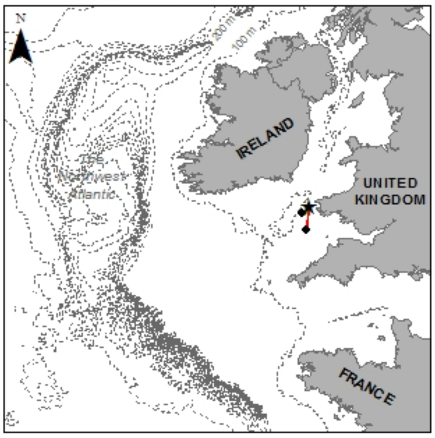

G424 - breeding female (2016, n = 2 trips)

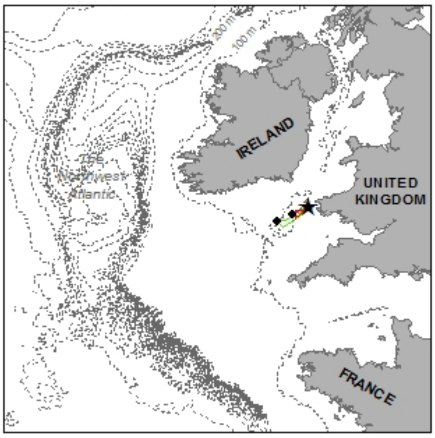

G435 - breeding female (2016, n = 2 trips)

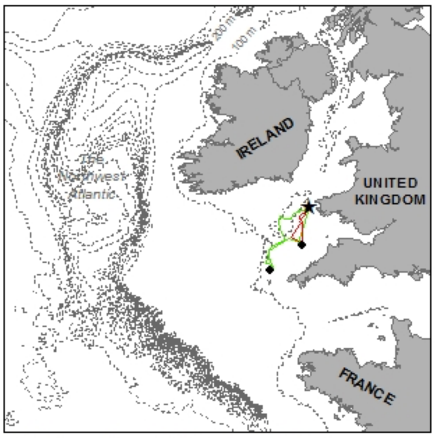

G441 - breeding male (2016, n = 2 trips)

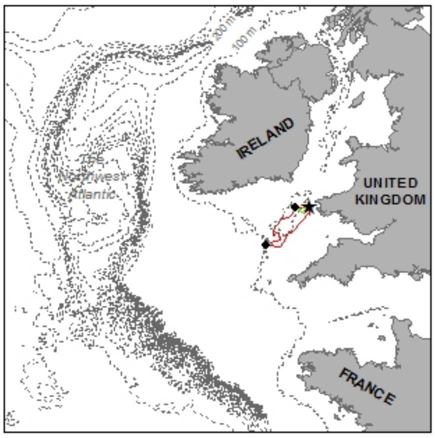

G444 - breeding male (2016, n = 2 trips)

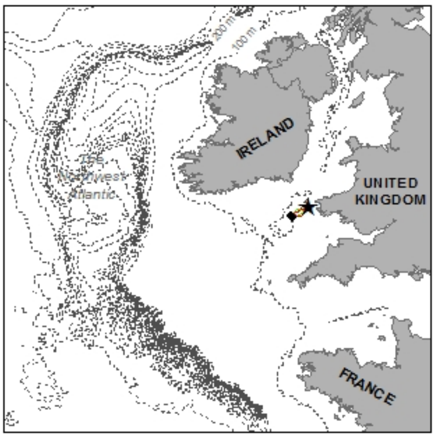

G447 - breeding male (2016, n = 2 trips)

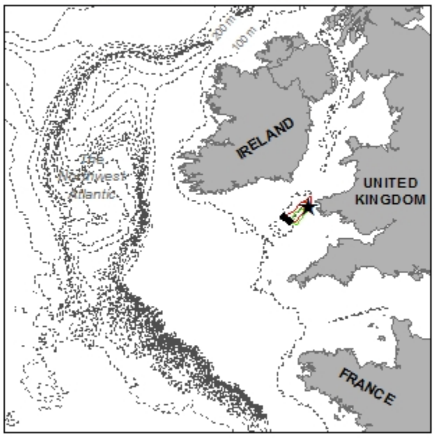

G450 - breeding female (2016, n = 2 trips)

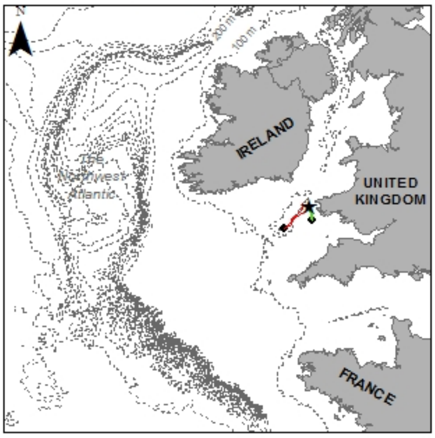

G458 - breeding female (2016, n = 2 trips)

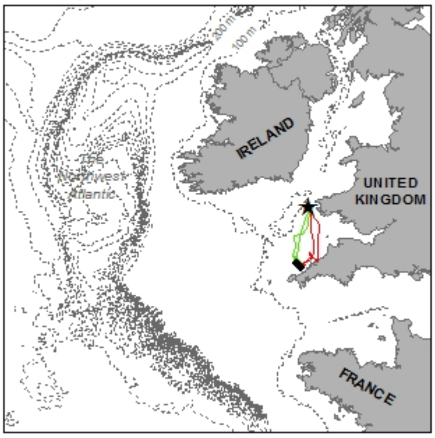

G461 - breeding male (2016, n = 4 trips)

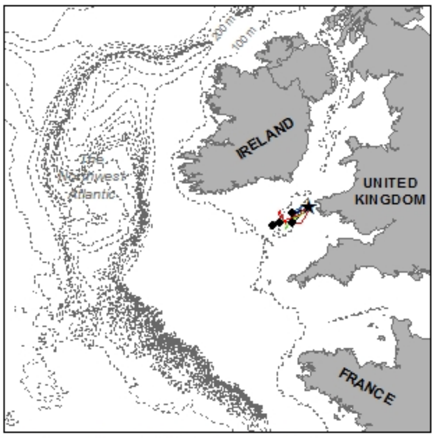

G464 - breeding male (2016, n = 3 trips)

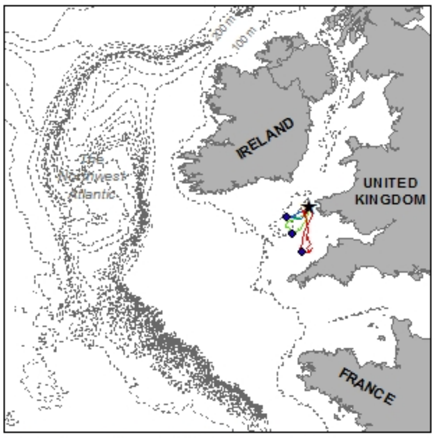

G466 - breeding male (2016, n = 2 trips)

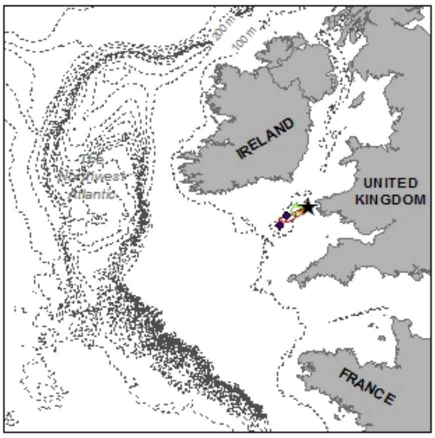

G469 - breeding male (2016, n = 5 trips)

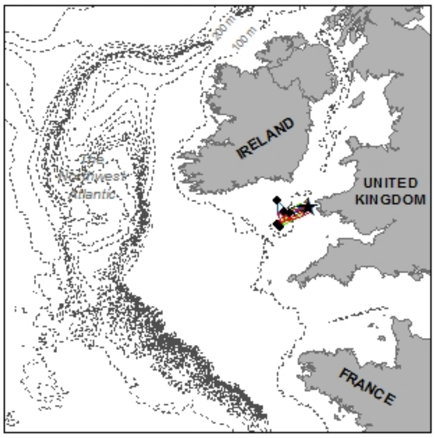

G415 - breeding male (2016, n = 4 trips)

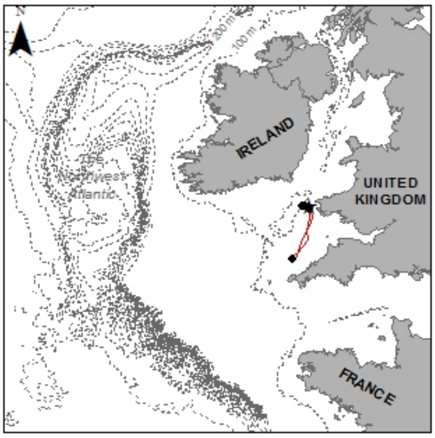

G434 - breeding male (2016, n = 2 trips)

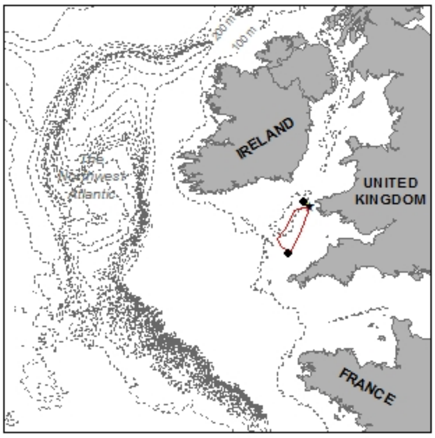

G446 - breeding female (2016, n = 2 trips)

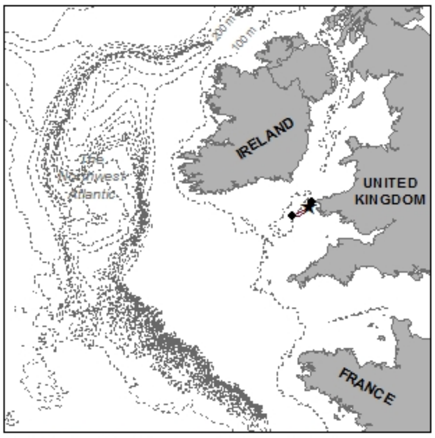

G451 - breeding female (2016, n = 2 trips)

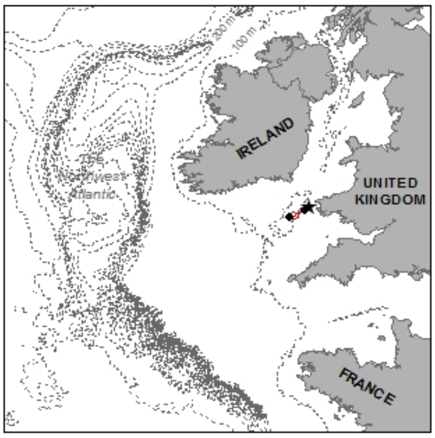

G454 - breeding female (2016, n = 2 trips)

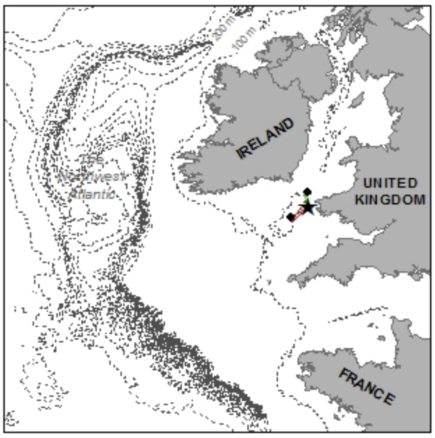

G011 - breeding male (2010, n = 5 trips)

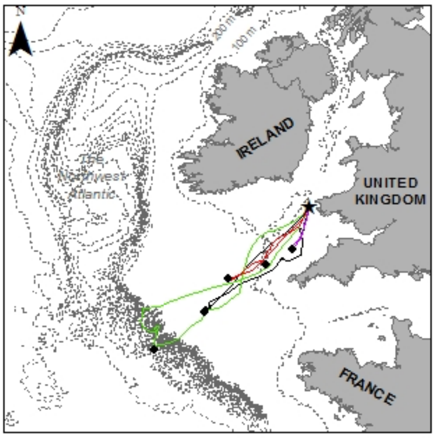

G019 - breeding female (2010, n = 8 trips)

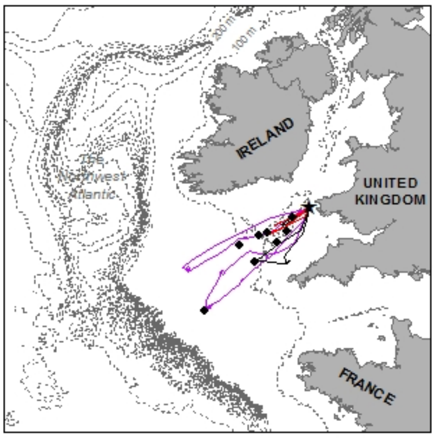

G368 - breeding male (2015, n = 2 trips)

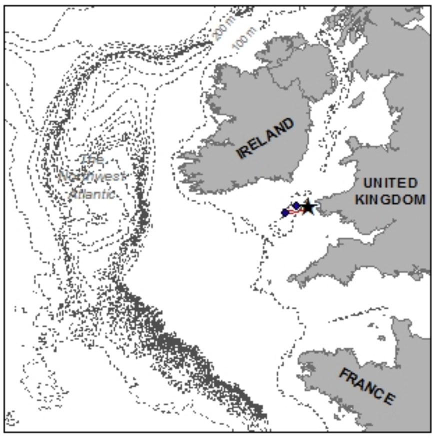

G359 - breeding female (n = 2 trips)

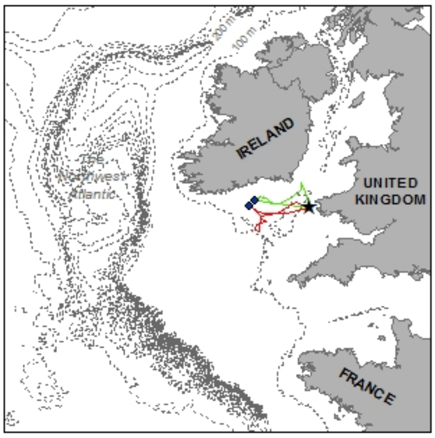

G372 - breeding male (2015, n = 3 trips)

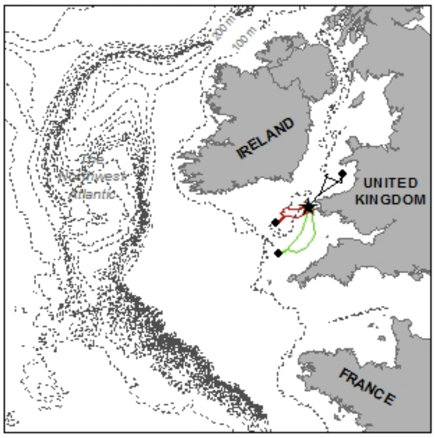

Electronic supplementary material, figure 3. Repeat central place foraging tracks of (n=15) immature northern gannets (aged 2-3 years) from Grassholm, UK, July and August 2010, 2015 and 2016 generated from GPS-PTT and GPS-GSM tags. Tracks are colour-coded by number: track 1=black; track 2=blue; track 3=red; track 4 = green; track 5 = purple. Data is presented for 8 males and 7 females.

57619 - immature female (2010, n = 4 trips)

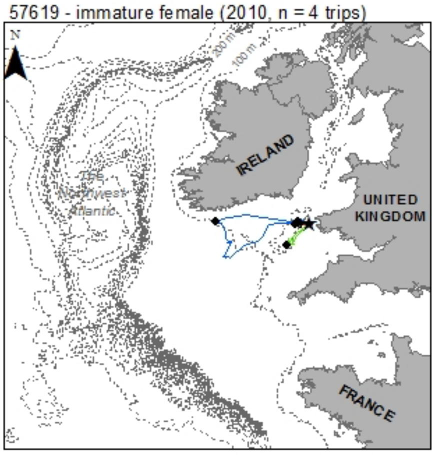

57629 - immature female (2010, n = 4 trips)

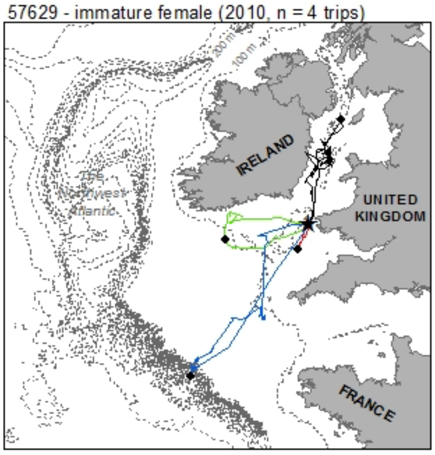

57621 - immature female (2010, n = 4 trips)

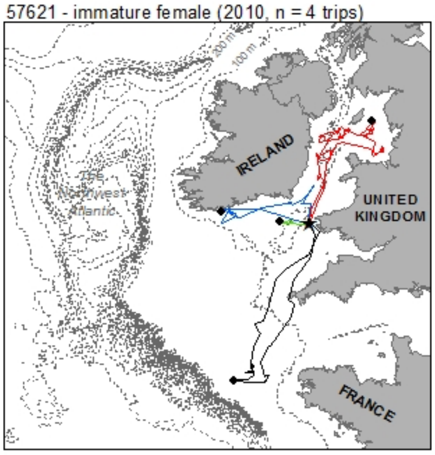

57628 - immature female (2010, n = 4 trips)

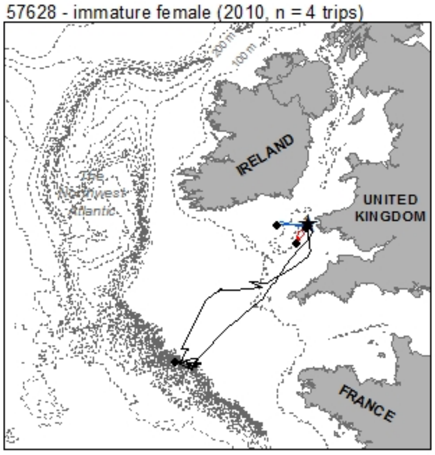

57631 - immature female (2010, n = 4 trips)

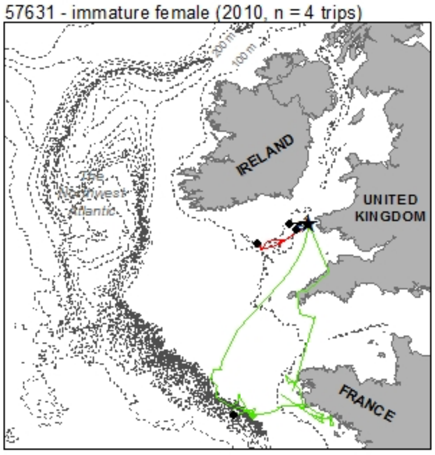

57633 - immature female (2010, n = 4 trips)

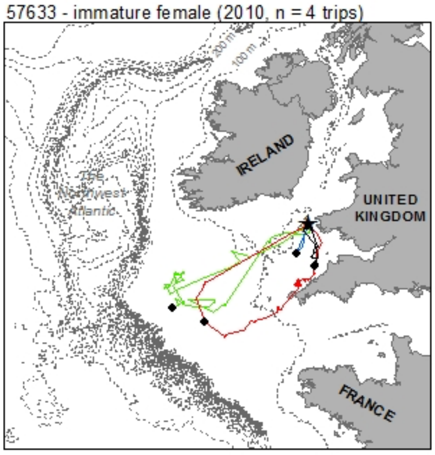

57634 - immature male (2010, n = 4 trips)

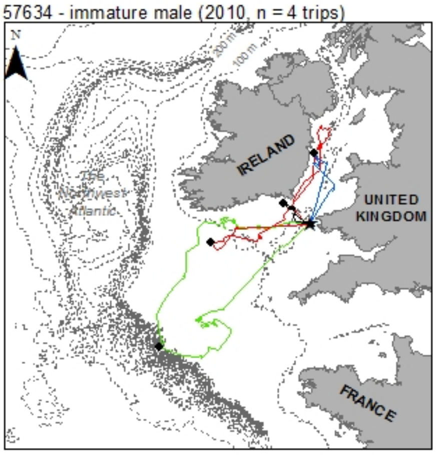

57643 - immature male (2010, n = 2 trips)

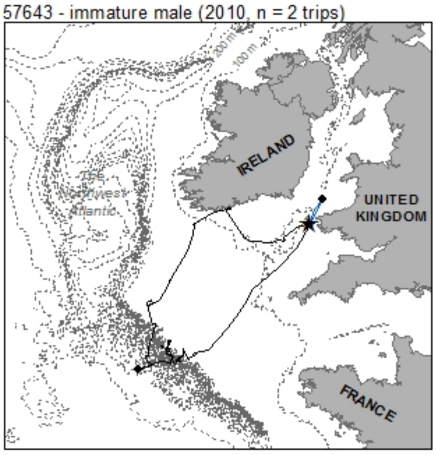

G394 - immature male (2015, n = 4 trips)

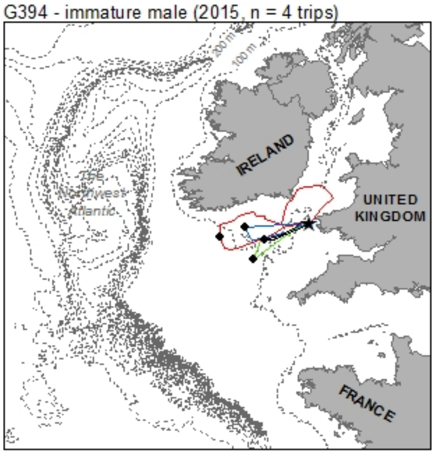

G395 - immature female (2015, n = 5 trips)

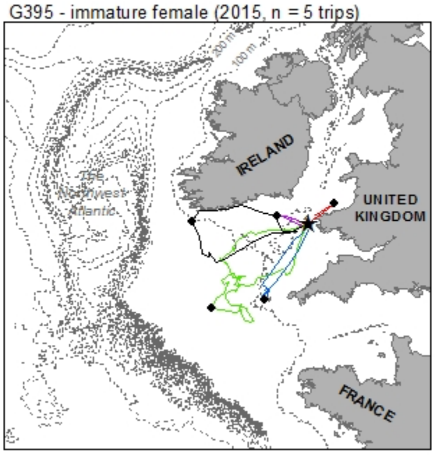

G401 - immature male (2015, n = 3 trips)

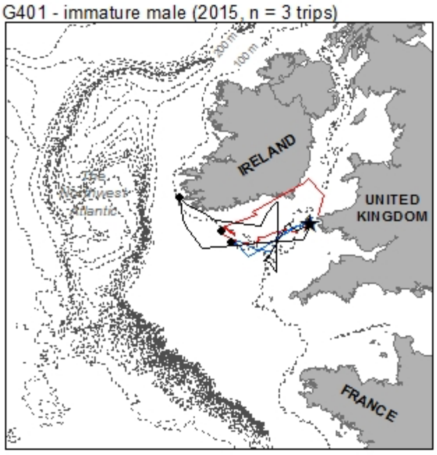

G407 - immature female (2015, n = 4 trips)

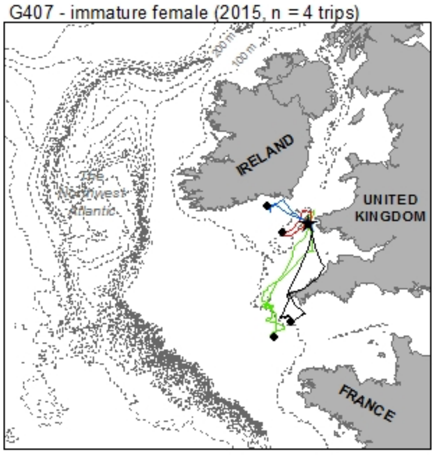

G429 - immature male (2016, n = 5 trips)

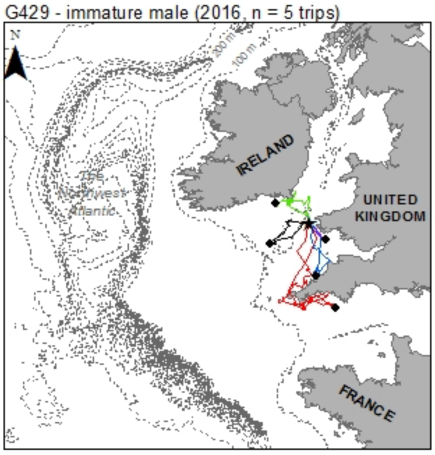

G430 - immature male (2016, n = 7 trips)

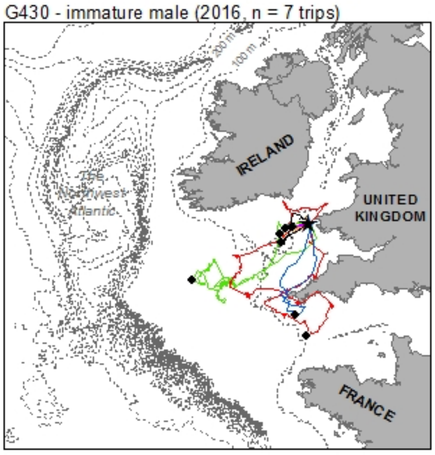

G433 - immature male (2016, n = 8 trips)

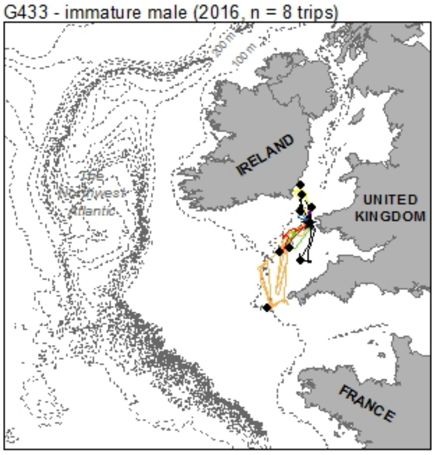

Electronic supplementary material, figure 4. Repeat central place foraging tracks of (n=5) failed or sabbatical breeding northern gannets from Grassholm, UK, July 2015. Tracks are colour-coded by number: track 1=black; track 2=blue; track 3=red; track 4 = green.

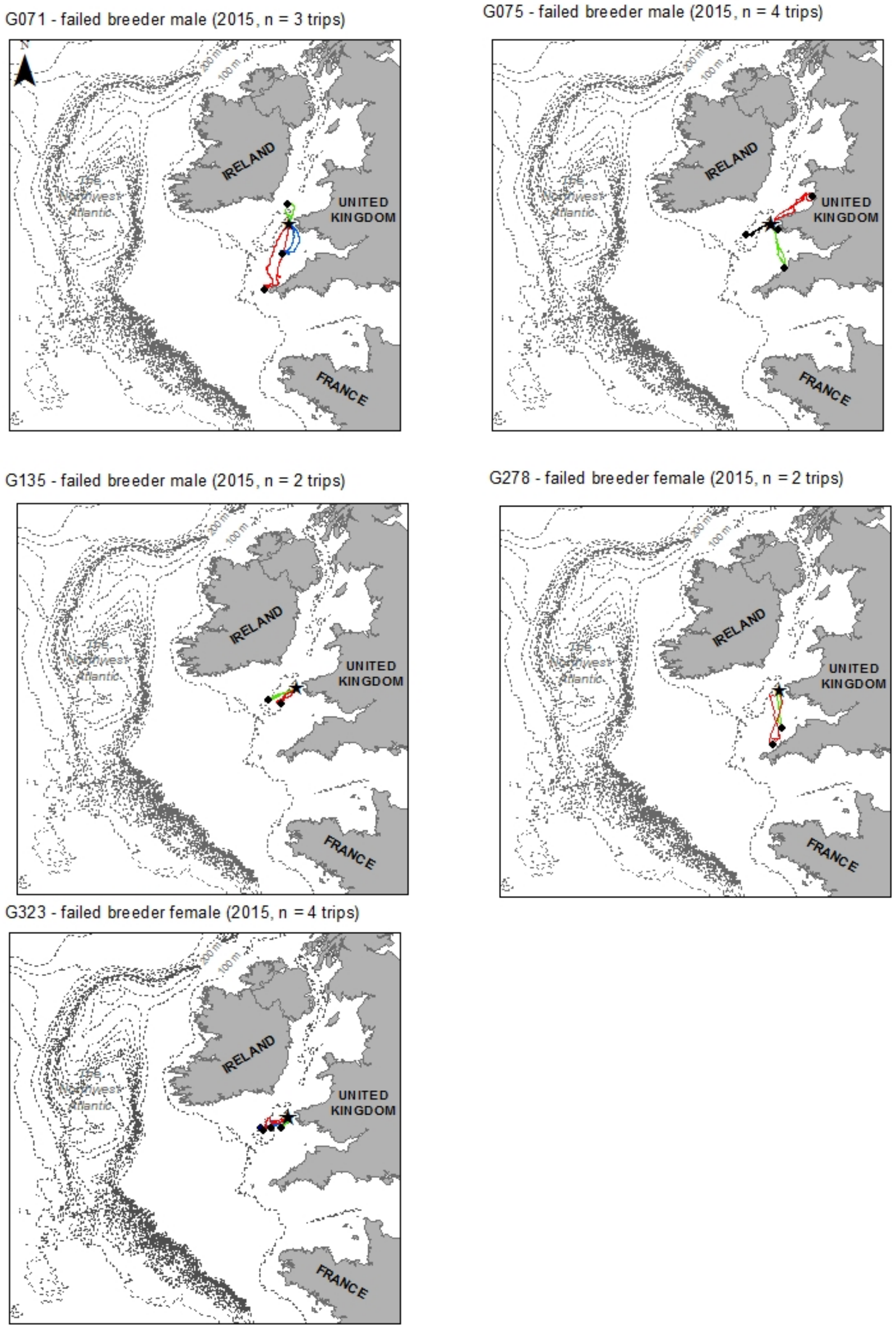

Supplement: Table of tracking data; Plots of NND sex differences; Breeder tracking data; Immature tracking data; Failed breeder tracking data [file rspb20171068supp1.pdf]
